# Examining treatment responses of diagnostic marrow in murine xenografts to predict relapse in children with acute lymphoblastic leukaemia

**DOI:** 10.1038/s41416-020-0933-4

**Published:** 2020-06-15

**Authors:** Abdulmohsen M. Alruwetei, Katerina Bendak, Babasaheb D. Yadav, Hernan Carol, Kathryn Evans, Chelsea Mayoh, Rosemary Sutton, Glenn M. Marshall, Richard B. Lock

**Affiliations:** 1 Children’s Cancer Institute, School of Women’s and Children’s Health, UNSW Sydney, Sydney, NSW Australia; 2 College of Applied Medical Science, Medical Laboratory Department, Qassim University, Qassim, Saudi Arabia; 3 Kids Cancer Centre, Sydney Children’s Hospital, Randwick, NSW Australia

**Keywords:** Experimental models of disease, Acute lymphocytic leukaemia, Cancer models

## Abstract

**Background:**

While current chemotherapy has increased cure rates for children with acute lymphoblastic leukaemia (ALL), the largest number of relapsing patients are still stratified as medium risk (MR) at diagnosis (50–60%). This highlights an opportunity to develop improved relapse-prediction models for MR patients. We hypothesised that bone marrow from MR patients who eventually relapsed would regrow faster in a patient-derived xenograft (PDX) model after induction chemotherapy than samples from patients in long-term remission.

**Methods:**

Diagnostic bone marrow aspirates from 30 paediatric MR-ALL patients (19 who relapsed, 11 who experienced remission) were inoculated into immune-deficient (NSG) mice and subsequently treated with either control or an induction-type regimen of vincristine, dexamethasone, and *L*-asparaginase (VXL). Engraftment was monitored by enumeration of the proportion of human CD45^+^ cells (%huCD45^+^) in the murine peripheral blood, and events were defined a priori as the time to reach 1% huCD45^+^, 25% huCD45^+^ (TT25%) or clinical manifestations of leukaemia (TTL).

**Results:**

The TT25% value significantly predicted MR patient relapse. Mutational profiles of PDXs matched their tumours of origin, with a clonal shift towards relapse observed in one set of VXL-treated PDXs.

**Conclusions:**

In conclusion, establishing PDXs at diagnosis and subsequently applying chemotherapy has the potential to improve relapse prediction in paediatric MR-ALL.

## Background

Acute lymphoblastic leukaemia (ALL) is the most common paediatric malignancy, accounting for approximately 20% of childhood cancers worldwide.^1^ Despite improvements in cure rates over the past half-century, ALL remains one of the most common causes of death from disease in childhood.^2^ When first presenting with the disease, children are assessed for clinical, molecular and biological features, and subsequently stratified according to their predicted risk of relapse into standard risk (SR), medium risk (MR) or high risk (HR) subgroups.^3–5^ This classification informs the selection of an appropriate treatment strategy, whereby those stratified into the SR and MR subtypes are treated with less intensive chemotherapy, while more aggressive protocols are reserved for those exhibiting an HR subtype.^6–8^

Despite receiving risk-adapted therapy, 15–20% of ALL patients will subsequently relapse at a median of 26 months from diagnosis. The largest number of relapsing patients occurs among MR-ALL patients, since this is the largest risk group (with 60–70% of patients) and has 20–22% relapse rate compared to 7−11% relapses in SR patients at 7 years.^5,9^ The first clinical trials that substituted minimal residual disease (MRD) assessment for NCI Rome criteria to define high-risk patients showed improved outcomes for their small HR groups,^7,9^ but had larger MR groups.^5,10,11^ While additional molecular risk criteria have been developed and are being implemented in current cohorts, including day 15 MRD, the *P2RY8-CRLF2* fusion and *IKAROS* (*IKZF1*) deletions or Ph-like disease,^12–16^ these do not identify all potential B-cell precursor (BCP) ALL relapses. Consequently, other methods of predicting patient relapse based on whole-genome sequencing (WGS) or patient-derived xenografts (PDXs) are worthy of investigation.

PDXs, which are created by transplantation of patient cancer cells into immune-deficient mice, currently provide the most clinically relevant experimental model to study in vivo response to chemotherapy and disease progression in paediatric ALL.^17,18^ ALL PDXs closely resemble the morphology, phenotype and genotype of the original tumour,^19–22^ can reflect the responses of patients to their treatment,^23,24^ and in some instances can predict clinical outcome.^25–27^ Recent advances in next-generation sequencing enable the detailed examination of genomic profiles to study the role of genetic defects driving paediatric leukaemia,^28^ and in particular to identify potential causative forces associated with relapse.^29–31^

The aim of this study was to test whether the engraftment characteristics of MR-ALL samples exposed to an induction-type chemotherapy regimen could predict relapse in MR-ALL patients. Here we show that treatment response characteristics of diagnostic MR patient marrows grown as a murine PDX were highly specific for relapse indicating clinical utility.

## Methods

### Patient samples

The study was conducted on bone marrow (BM) biopsy samples of patients who were enrolled in the Australia and New Zealand Children’s Haematology and Oncology Group (ANZCHOG) ALL Study VIII clinical trial and completed their treatment according to the guidelines of the protocol.^7^ All patients selected for this study were MR patients diagnosed with BCP-ALL with bio-banked vials of diagnosis mononuclear cells. Patients with >90% blasts in the BM were selected for this study (with the exception of ALL-215, for which the per cent blasts was unknown). The MR cohort excluded patients who were MRD-negative by day 33 (SR) and patients who had either high MRD at week 12 (>5 × 10^−4^), poor prednisone response, or *BCR-ABL1* or *MLL* rearranged (HR). The study initially compared three groups of patients based on survival data, defined as patients who relapsed on treatment (*n* = 9), off treatment (*n* = 10) (Rel) and patients in complete remission (CR1) with >7 years follow-up (*n* = 11). Since there was no apparent difference between the two relapse groups, these were combined for analyses. All patient details are listed in Table 1.

**Table 1:** Clinical characteristics of MR ALL patient samples.

PDX	Outcome group	Sex	Age at diagnosis (years)	WCC (10^9^/l)	Cytogenetics	Length of CR1 (months)	Site of relapse	MRD (day 15)	MRD (day 33)	MRD (day 79)	Status at last follow-up
ALL-65/220	CR1	F	2	9.8	Hyperdiploid > 50	>84	—	<1 × 10^–4^	<1 × 10^–4^	negative	CR1 > 7 years
ALL-67	CR1	F	2.3	166.9	ETV6-RUNX1	>98	—	<1 × 10^–4^	N/A	N/A	CR > 8 years
ALL-206	CR1	M	4.4	6.6	ETV6-RUNX1	>120	—	>1 × 10^–1^	<1 × 10^–3^	<1 × 10^–4^	CR1 > 10 years
ALL-207	CR1	M	12.6	7.8	B other	>84	—	<1 × 10^–4^	<1 × 10^–3^	<1 × 10^–4^	CR1 > 7 years
ALL-208	CR1	M	12.7	50.3	B other	>74	—	>1 × 10^–1^	<1 × 10^–2^	<1 × 10^–4^	CR1 > 6 years
ALL-212	CR1	M	6.3	3.2	Hyperdiploid > 50	>120	—	<1 × 10^–2^	<1 × 10^–3^	<1 × 10^–4^	CR1 > 10 years
ALL-213	CR1	F	3	158.4	B other	>108	—	<1 × 10^–2^	<1 × 10^–4^	Negative	CR1 > 9 years
ALL-214	CR1	M	6.4	45.8	B other	>120	—	<1 × 10^–1^	<1 × 10^–3^	Negative	CR1 > 10 years
ALL-223	CR1	F	3.6	2.6	Unclear	>108	—	<1 × 10^–1^	<1 × 10^–3^	Negative	CR1 > 9 years
ALL-224	CR1	F	8.5	22	B other	>120	—	>1 × 10^–1^	<1 × 10^–3^	Negative	CR1 > 10 years
ALL-231	CR1	F	5.1	20.1	B other	>108	—	N/A	< 1 × 10^–4^	Negative	CR1 > 9 years
ALL-232	CR1	M	2.9	30.7	ETV6-RUNX1	>96	—	<1 × 10^–3^	<1 × 10^–4^	Negative	CR1 > 8 years
ALL-64/202	Rel	F	1.6	3.1	Hyperdiploid > 50	19	BM	N/A	< 1 × 10^–4^	<1 × 10^–4^	DOD
ALL-66/215	Rel	F	2.4	135.8	ETV6-RUNX1	19	BM	<1 × 10^–4^	<1 × 10^–4^	Negative	Alive in CR2
ALL-201	Rel	F	16.7	11.2	B other	25	BM	<1 × 10^–3^	<1 × 10^–3^	<1 × 10^–4^	Alive in CR2
ALL-203	Rel	M	17.3	47.7	B other	20	BM	<1 × 10^–4^	Negative	Negative	DOD
ALL-209	Rel	M	12.9	22.2	B other	29	BM/CNS	<1 × 10^–2^	<1 × 10^–4^	<1 × 10^–4^	Alive in CR2
ALL-210	Rel	F	13.5	4.5	Hyperdiploid > 50	24	BM	>1 × 10^–1^	<1 × 10^–3^	<1 × 10^–4^	TRM
ALL-211	Rel	M	3.4	9.4	Hyperdiploid > 50	24	CNS	<1 × 10^–1^	<1 × 10^–2^	Negative	DOD
ALL-216	Rel	M	8.7	34.9	B other	17	CNS	N/A	<1 × 10^–2^	Negative	DOD
ALL-217	Rel	M	12.2	4.5	TCF3-PBX1	24	BM	N/A	<1 × 10^–4^	<1 × 10^–4^	Alive in CR2
ALL-218	Rel	F	6.5	21.1	Hyperdiploid > 50	36	BM	N/A	<1 × 10^–1^	<1 × 10^–3^	Alive in CR2
ALL-219	Rel	M	15.7	9.7	B other	25	CNS	N/A	N/A	<1 × 10^–3^	DOD
ALL-221	Rel	M	3.9	38.3	ETV6-RUNX1	38	BM	<1 × 10^–2^	<1 × 10^–3^	<1 × 10^–3^	Alive in CR2
ALL-222	Rel	F	7.6	33.4	ETV6-RUNX1	35	BM	<1 × 10^–1^	<1 × 10^–3^	Negative	Alive in CR2
ALL-225	Rel	F	10.8	2.6	Hyperdiploid > 50	38	BM	<1 × 10^–1^	<1 × 10^–2^	Negative	Alive in CR2
ALL-226	Rel	F	5.2	12.4	B other	30	BM	N/A	<1 × 10^–3^	Negative	Alive in CR2
ALL-227	Rel	M	3.1	70.9	B other	28	BM/CNS/testes	<1 × 10^–1^	<1 × 10^–2^	<1 × 10^–4^	Alive in CR4
ALL-228	Rel	M	6	27.5	ETV6-RUNX1	66	BM	<1 × 10^–1^	<1 × 10^–2^	<1 × 10^–4^	Alive in CR2
ALL-229	Rel	F	11.2	4.6	B other	31	BM/CNS	N/A	Negative	<1 × 10^–4^	DOD
ALL-230	Rel	F	8.2	428.1	B other	27	CNS	<1 × 10^–1^	<1 × 10^–2^	Negative	Alive in CR3
—	—	*P* = 0.89	*P* = 0.09	*P* = 0.88	*P* = 0.73	*P* = 0.00	—	*P* = 0.80	*P* = 0.48	*P* = 0.15	—

*PDX* patient-derived xenograft, *CR1/2/3/4* first/second/third/fourth complete remission, *Rel* relapse, *F* female, *M* male, *WCC* white cell count, *BM* bone marrow, *CNS* central nervous system, *MRD* minimal residual disease, *N/A* not available, *DOD* died of disease, *TRM* transplantation-related mortality.

### PDX model in immune-deficient mice

ALL PDXs were established from previously cryopreserved MR BCP-ALL BM aspirates from patients at diagnosis. Female nonobese diabetic/severe combined immunodeficient (NOD.CB17-*Prkdc*^*scid*^/SzJ, NOD/SCID) or NOD/SCID common cytokine receptor gamma chain^–/–^ (NOD.Cg-*Prkdc*^*scid*^ *Il2rg*^*tm1Wjl*^/SzJAusb, NSG) mice were housed in a specific pathogen free facility on a 12 h/12 h light/dark cycle with a minimum of two and maximum of six animals per translucent amber polycarbonate autoclavable cage (Tecniplast, Italy) with air filters and dimensions of 14 cm W × 21 cm H × 27 cm L, or 22 cm W × 15 cm H × 30 cm L). Additional enrichment was provided by including red polycarbonate igloos in each cage, and nesting material which allows for species-specific behaviour. Animals of 5–9 weeks of age and 20–25 g were inoculated with 1 × 10^6^ viable patient BM cells either via the lateral tail vein (intravenous, i.v.) or via the femur (intrafemoral, i.f.). Two weeks post inoculation, mice were randomised to receive either VXL treatment (vincristine, 0.15 mg/kg every 7 days for 2 weeks; dexamethasone, 5 mg/kg Mon−Fri for 2 weeks; *L*-asparaginase 1000 IU/kg Mon−Fri for 2 weeks) or vehicle-control (saline) via intraperitoneal injection. Leukaemia progression was monitored by weekly enumeration of the proportion of human CD45^+^ cells in the peripheral blood (%huCD45^+^) as described previously.^19,23^ Mouse engraftment was assessed a priori using three criteria: time to 1%huCD45^+^ (TT1%); time to 25%huCD45^+^ (TT25%); and time to overt symptoms of leukaemia (time to leukaemia, TTL).^27^ The endpoint of the experiment was mouse engraftment or a maximum holding time of 40 weeks. In addition, animals were monitored daily for signs of stress and/or pain. A pre-formulated key was followed to monitor signs of distress (includes posture and mobility, activity, appetite, appearance and grooming, hydration, colour of limbs, colour of mucosae, etc.). Weight monitoring was scheduled weekly with additional weight checks performed if indicated. At the end of each experiment mice were killed by CO_2_ asphyxiation according to the “UNSW guidelines on the use of carbon dioxide as a method of euthanasia for laboratory mice and rats”.

### Statistical analysis

Student’s *t* test or Fisher’s exact test were used to compare patient data. Kaplan−Meier survival curves to compare mouse and human event-free survival (EFS) were analysed using the exact log-rank test (GraphPad Prism 7.04 for Windows, GraphPad Software, La Jolla California USA). Receiver operating characteristic (ROC) analysis to determine the optimal data distribution was conducted using MedCalc Software (v18.11; Ostend, Belgium) available online at www.medcalc.org/calc/diagnostic_test.php. For all statistical tests, the level of significance was set at *P* < 0.05.

### Next-generation sequencing analysis

Paired-end WGS was conducted at the Beijing Genomics Institute (BGI, China) on diagnostic, remission and relapse biopsies. Targeted amplicon sequencing was also carried out on these samples and corresponding PDXs. Further details can be found in Supplementary Methods.

## Results

### Optimising engraftment conditions for a PDX model

Several strains of immune-deficient mice are receptive to engraftment of ALL under different conditions.^23,25,26,32^ Initially, the optimal experimental conditions to develop a PDX model for outcome prediction in paediatric MR ALL were assessed in a pilot study that used biopsy samples obtained at diagnosis from two pairs of patients, each with similar clinical and disease characteristics but with different clinical outcomes (ALL-64, relapse at 19 months (Rel) and died of disease (DOD); ALL-65, in prolonged complete remission (CR1 for ≥84 months); ALL-66, relapsed at 19 months (Rel); and ALL-67 (CR1 for ≥98 months), Supplementary Table S1). These were inoculated into groups of four immune-compromised mice using combinations of different engraftment variables (mouse strain, NSG versus NOD/SCID; route of inoculation, i.f. versus i.v.; chemotherapy, VXL versus saline-control; Supplementary Table S2) (total 128 mice). Weekly monitoring of engraftment kinetics in these PDXs showed that: (1) the engraftment of primary ALL cells showed improved efficiency in NSG mice (49/63 mice engrafted, 78%) compared to NOD/SCID mice (34/64 mice engrafted, 53%); (2) there was improved engraftment efficiency following i.v. (45/64 mice engrafted, 70%) compared to i.f. (38/63 mice engrafted, 60%) inoculation of cells; and (3) VXL treatment of mice reduced efficiency of engraftment overall (vehicle-control: 49/64 mice engrafted, 77%; VXL-treated: 34/63 mice engrafted, 54%; Supplementary Fig. S1 and Supplementary Table S2). However, none of the observed differences were statistically significant using Student’s *t* test (NSG compared to NOD/SCID: *P* = 0.07; i.v. compared to i.f.: *P* = 0.50; saline-control compared to VXL: *P* = 0.09). The engraftment conditions selected for the main study were NSG mice inoculated via the i.v. route with subsequent VXL or saline treatment.

### Establishment of a PDX model for outcome prediction in paediatric MR-ALL

To test whether a PDX model could be used to predict patient relapse, the main study was conducted using 30 MR BCP-ALL patients selected from the ANZCHOG ALL8 trial (Supplementary Fig. S2).^7^ Only patients with high numbers of bio-banked vials were used, resulting inadvertently in a higher proportion of patients with NCI (National Cancer Institute) high-risk characteristics (>10 years old or white cell counts > 50). A total of 11 patients who remained in complete remission (CR1) for >72 months and 19 patients who experienced relapse (Rel) prior to 72 months from diagnosis were compared (Table 1). The 19 relapsed cases included 9 relapses that occurred on therapy and 10 off-therapy relapses (including repeats of ALL-64 and ALL-66 which were first engrafted in the pilot study). The 11 patients in long-term remission included 10 with >72 months follow-up and a repeat of ALL-65 (first engrafted in the pilot study). The order of engraftment of patients was randomised and the researcher performing the engraftment studies was blinded for patient outcome until completion of the study. For each patient sample, six NSG mice were inoculated intravenously with 1 × 10^6^ patient BM cells. Two weeks later, the six mice were randomised to receive either VXL (*n* = 3) or saline vehicle-control treatment (*n* = 3) for 2 weeks, following which mice were monitored and disease progression determined as described in the “Methods” section (total 180 mice).

**Fig. 1:**
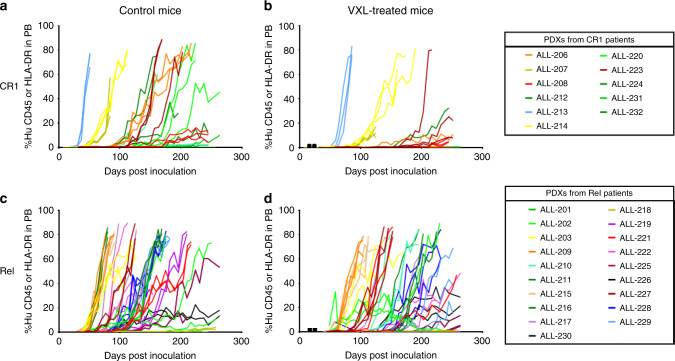
Compiled engraftment data of the cohort of 30 MR ALL patient samples used in the main study. Graphs show the peripheral blood progression patterns of primary samples inoculated into control (**a**, **c**) and VXL-treated (**b**, **d**) mice to establish PDXs from patients who experienced CR1 (**a**, **b**) and Rel (**c**, **d**). Black squares on the *x*-axis indicate VXL treatment points.

Overall, 93% (28/30) of diagnosis samples engrafted in 77% (138/180) of inoculated mice over the monitoring period of 9 months, with variability between patients in the kinetics and levels of leukaemia dissemination into the murine peripheral blood (Fig. 1 and Supplementary Table S3). The first control mouse showed evidence of engraftment (1%huCD45^+^) 24.3 days post inoculation, with the median of all control mice showing 1%huCD45^+^ by 81.3 days and the last mouse engrafted to 1%huCD45^+^ by 196.9 days post inoculation. Most patient samples exhibited consistent patterns of disease progression within their respective groups of three control or VXL-treated mice. Of note, while the engraftment kinetics of biopsies from both groups of patients (CR1 and Rel) did not differ significantly in saline-treated mice (Fig. 1a, c), VXL treatment markedly inhibited disease progression in mice inoculated from the CR1 group (Fig. 1b) but not the Rel group (Fig. 1d), warranting a more detailed investigation.

The reproducibility of the PDX model system was evaluated by comparing engraftments in the pilot study with those in the main study for the three repeated samples. The patterns of engraftment were remarkably consistent between each of the three pairs initially engrafted during the pilot study (ALL-64, ALL-65 and ALL-66) and re-engrafted during the main study (ALL-202, ALL-220 and ALL-215, respectively; Supplementary Fig. S3).

Overall, the engraftment data demonstrated that: (1) the i.v. engraftment of primary MR-ALL patient samples in NSG mice was highly efficient; (2) there was considerable heterogeneity in the kinetics of engraftment between patients and between CR1 and Rel groups; and (3) VXL treatment delayed the re-emergence of ALL more effectively in the CR1 group. Next, the engraftment characteristics of the two groups were examined to develop the most effective model for predicting relapse.

### Optimal PDX engraftment characteristics after VXL which distinguish CR1 and rel patients

**Fig. 2:**
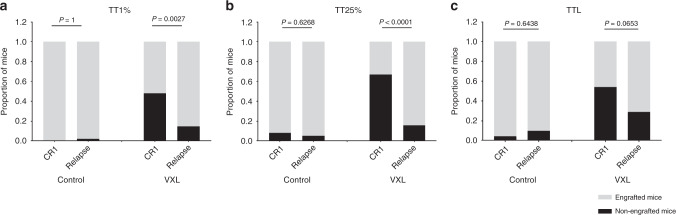
Comparison of the number of non-engrafted and engrafted mice using different regrowth characteristics. The engraftment of patient samples in mice is presented based on the number of non-engrafted and engrafted mice in the VXL-treated and saline-control groups at **a** TT1%, **b** TT25%, **c** TTL. Engraftment was compared for the relapse or CR1 patient groups as a whole, using Fisher’s exact test.

The CR1 and Rel groups were compared for several different measures of disease progression including time to 1%huCD45^+^ (TT1%), time to 25%huCD45^+^ (TT25%) and time to overt symptoms of leukaemia (time to leukaemia, TTL; Fig. 2). The PDXs in which none of the control mice reached event (two PDXs at TT1%, seven PDXs at TT25% and seven PDXs at TTL) were excluded from analysis, since their inclusion would not inform the relapse-prediction model.

**Fig. 3:**
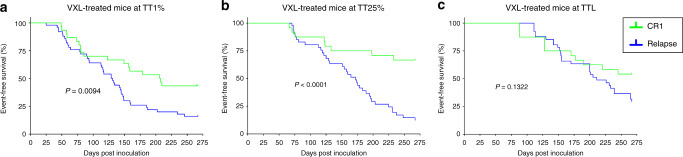
Comparison of mouse engraftment between CR1 and Rel-derived PDXs based on different timepoints in VXL-treated mice. The EFS of VXL-treated mice was defined as TT1% (**a**), TT25% (**b**) and TTL (**c**) for each patient outcome group (CR1 and Rel). The EFS of mice was plotted using Kaplan−Meier curves and compared for statistical significance using the log-rank test.

The mouse PDXs treated with saline (vehicle-control) showed no significant differences between the proportion of CR1 and Rel samples engrafted using any of the three engraftment criteria when applying the Fischer’s exact test (Fig. 2). However, a significantly lower proportion of CR1 samples engrafted compared with Rel in VXL-treated mice when engraftment was assessed at TT1% (*P* = 0.0027; Fig. 2a) and TT25% (*P* < 0.0001; Fig. 2b), but not for TTL (*P* = 0.065; Fig. 2c). These findings indicate that using VXL treatment may facilitate stratification of patients according to PDX outcome as early as TT1%, (i.e. at a median time of 129 (44–234) days after inoculation; Supplementary Table S3). In Fig. 3, CR1 and Rel groups were compared in Kaplan−Meier survival curves for either TT1%, TT25% or TTL values, showing that the CR1 VXL-treated PDX samples progressed significantly slower at TT1% (*P* = 0.0094, Fig. 3a) and TT25% (*P* < 0.0001, Fig. 3b), but not at TTL (*P* = 0.1322, Fig. 3c), compared to Rel VXL-treated PDXs.

Next, the ability of VXL treatment to differentiate CR1 and Rel samples was assessed using the median time of each group and the first mouse in each group to reach the event threshold. Using the median time to engraftment of each group also discriminated between CR1 and Rel patients (TT1%, *P* = 0.029, Supplementary Fig. S4A; TT25%, *P* = 0.0191, Supplementary Fig. S4C). When comparing the time taken for the first mouse of each group to reach the event threshold, the only significant difference observed between CR1 and Rel patients was at TT25% (*P* = 0.0189, Supplementary Fig. S4D). Neither TTL (not shown) nor TT1% (*P* = 0.2338, Supplementary Fig. S4B) were predictive. Taken together these results suggest that TT25% is the strongest discriminator of CR1 and Rel patients in VXL-treated mice.

### Determining the predictive value of the VXL-treated PDX model for relapse

**Fig. 4:**
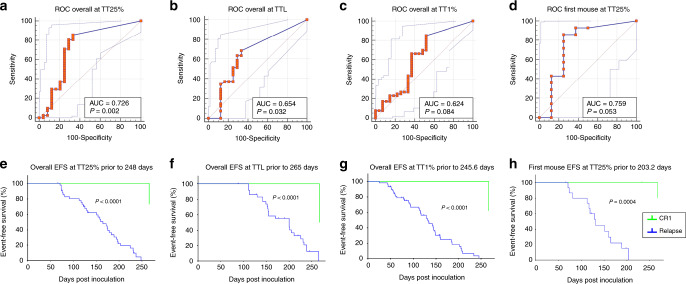
Development of a PDX model predictive of relapse in MR ALL. ROC analysis was conducted to determine the optimal criteria predicting patient outcome for individual mice reaching TT25% (**a**), individual mice reaching TTL (**b**), individual mice reaching TT1% (**c**) and the first mouse reaching TT25% (**d**). Kaplan−Meier graphs show the percentage of engrafted mice in the MR ALL cohort based on classifiers determined by ROC analysis of individual mice reaching TT25% prior to 248 days (**e**), individual mice reaching TTL prior to 265 days (**f**), individual mice reaching TT1% prior to 245.6 days (**g**) or the first mouse reaching TT25% prior to 203.2 days (**h**). The log-rank test was used to analyse statistical differences in length of time to disease progression between the two subgroups of patients.

To develop a tool capable of predicting patient outcome based on re-emergence of ALL in VXL-treated mice, ROC analysis was used to determine the optimal length of monitoring post inoculation for assessing regrowth of leukaemia in mice for the different variables (Supplementary Table S4, Fig. 4a–d). The ROC analyses, based on individual mice in each group, identified the best threshold for TT25% was ≤248 days (*P* = 0.002, Fig. 4a), for TTL ≤ 265 days (*P* = 0.032, Fig. 4b) and for mice reaching TT1% ≤ 245.6 days (*P* = 0.084, Fig. 4c). The best threshold when considering the first mouse of each group to reach TT25% was ≤203.2 days (*P* = 0.053, Fig. 4d). All other measurement criteria gave poorer discrimination (Supplementary Table S4 and Supplementary Fig. S5A−E). Next, the optimal thresholds from Fig. 4a–d were used to split the patients into two groups to generate Kaplan−Meier graphs for patient EFS (Fig. 4e–g). Thus, in Fig. 4e, patients whose VXL-treated PDXs had reached 25% huCD45^+^ cells in the peripheral blood by day 248 (blue line) had significantly more relapses than those patients whose ALL grew more slowly or not at all in the mice post VXL (green line; *P* < 0.0001). The other measures of disease progression in PDXs (TTL before 265 days or TT1% by 246 days or TT25% for first mouse) were also significant prognostic factors for patient relapse (*P* < 0.001%) (Fig. 4f–h). All other measurements of disease progression were less predictive (Supplementary Table S4 and Supplementary Fig. 5F−J).

To determine if subdividing the above samples into specific cytogenetic groups could influence, or even improve, the predictive power of the PDX model, the same analysis as above was performed for patients classified as *ETV6-RUNX1*, hyperdiploidy or B-other. Only the *ETV6-RUNX1* patient samples showed consistently significant differences in disease progression after VXL treatment for individual mice across all timepoints (TT1% before 159 days, TT25% before 237 days and TTL before 239 days; Supplementary Table S4), as well as median (TT1% before 159.8 days and TT25% before 224.6 days) and first mouse (TT1% before 90.8 days and TT25% before 188.8 days). The hyperdiploid subgroup only reached significance at TT25% before 231 days, whereas TT1% and TTL were only significant at the endpoint of the experiment (267 days). The B-other subgroup did not distinguish between patients in CR1 or those who experienced relapse.

Comparing ROC curves for individual mice, the median mouse and the first mouse to reach TT1%, TT25% and TTL, the best predictive model overall was to monitor all three VXL-treated mice, inoculated from an individual patient, to TT25% ≤ 248 days. This model distinguished between CR1 and Rel patients with 85.4% specificity (95%CI = 0.71–0.94) and 66.7% sensitivity (95%CI = 0.45–0.84; Supplementary Table S4). The large variation observed for sensitivity of this model may have been due to the small size of the cohort (*n* = 23), which exhibited two false-positive predictors showing fast disease progression into mice despite originating from the CR1 group of patients (Fig. 1b).

**Fig. 5:**
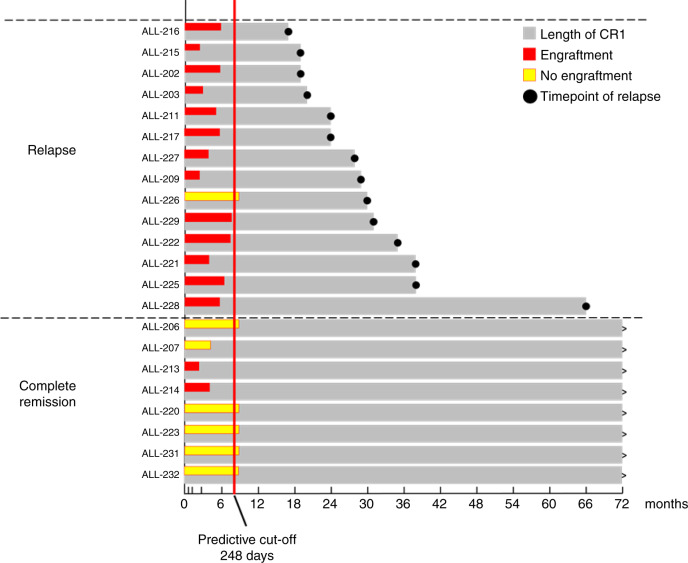
Timeline of patient outcome compared to PDX outcome. The length of patient CR1 and the engraftment time for the median of VXL-treated mice to reach TT25% are presented on the same timescale. Grey bars show the length of patient complete remission, with ongoing CR1 (black arrows) and timepoint of relapse (black dots) indicated. Median mouse engraftment is also shown (Red bar: median mouse reached event, yellow bar: mice never reached event). The predicted cut-off time determined by ROC analysis for all mice to reach TT25% is indicated (red vertical line).

The timeframes from diagnosis to clinical relapse in our study cohort relative to the time taken to evaluate their PDX samples were also examined (Fig. 5). All clinical relapses occurred well after the 248-day threshold (8.3 months), confirming that the model provides clinically relevant information which could be used to intensify therapy prior to clinical relapse in patients predicted to be at high risk of relapse.

We also compared the prediction of relapse in the informative PDXs (median TT25% greater or less than 248 days) to more standard methods of risk prediction in the entire cohort of 30 patients using log-rank (Mantel Cox) test and Kaplan−Meier plots. The PDX predictor was prognostic for relapse-free survival (RFS, *P* = 0.029, Supplementary Fig. 6A) but NCI risk was not significant (*P* = 0.074, Supplementary Fig. S6B). Other factors including: MRD status; *ETV6-RUNX1*; ALL favourable versus intermediate risk genetics; IKZF1plus status; Ph-like ALL status; IKZF1 deletion alone; and IKZF1 or CRLF2 deletions were also not significant (Supplementary Fig. S6C−I).

### Assessing molecular features in patient biopsies and corresponding PDXs

To test if the PDXs engrafted from diagnostic patient biopsies into mice reconstitute the patient’s molecular profile, patient samples and their corresponding PDXs underwent mutational analysis. Four MR BCP-ALL patients (corresponding PDXs: ALL-66/ALL-215, ALL-217, ALL-64/ALL-202 and ALL-203), exhibiting on-treatment relapse in the clinic and varying cytogenetic profiles (Table 1), were analysed using WGS from patient biopsies received at diagnosis, remission (MRD-negative) and relapse. Single nucleotide variant (SNV) analysis was performed as described in the “Methods” section with the median mutation burden being 1.79 Mut/Mb (range 0.46–10.56; Supplementary Table S5).

To validate the initial sequencing outcome, a total of 184 SNVs were selected from the four patient’s biopsies. SNVs were chosen to represent each of the patients at Dx only, Rel only or both, and included a wide range of variant allele frequencies (VAFs; 0.08–0.94) predicted to be either pathogenic or likely pathogenic. The SNV locations were then used to design a targeted sequencing panel (AmpliSeq, Illumina) which was applied to the patient samples together with their corresponding in vivo control and VXL-treated PDXs (Supplementary Table S6).

**Fig. 6:**
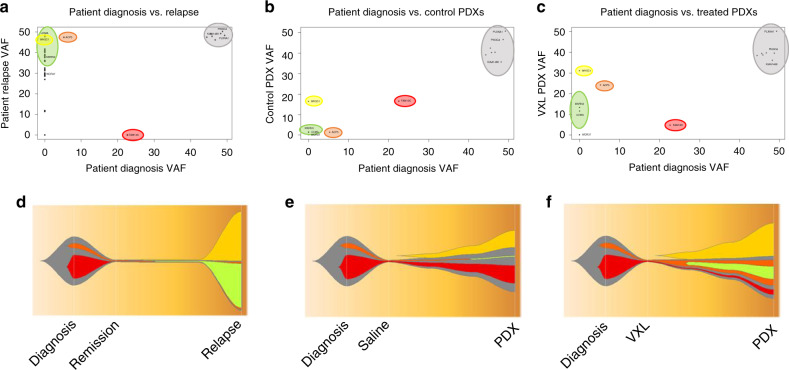
Sub-clonal analysis in ALL-215 patient biopsies and PDX samples. Scatter plots show comparison of variant allele frequency (VAF) of ALL-215 patient’s diagnosis (Dx) and relapse (Rel) biopsies (**a**), Dx and control PDXs (**b**) and Dx and VXL-treated PDXs (**c**). Five major sub-clones are highlighted for comparison between the conditions. Fishplots show inferred progression of sub-clonal evolution comparing patient’s Dx and Rel sample (**d**), Dx and control PDXs (**e**) and Dx and VXL-treated PDXs (**f**). The colours correspond to the sub-clones highlighted in graphs (**a**−**c**).

Twelve patient samples (Dx, Rem and Rel from four patients) together with 25 derived PDXs were sequenced using a custom made AmpliSeq (Illumina) panel, with an average coverage of >1000 reads. This resulted in 139/184 (76%) of the SNVs confirmed across the patient samples with a VAF > 0.05 (Supplementary Table S6). Strikingly, ALL-215 showed changes in the original diagnostic pattern of SNVs upon engraftment and VXL treatment (Fig. 6 and Supplementary Fig. S7). SNVs found in both Dx and Rel samples were retained (grey), whilst SNVs identified only at relapse (Fig. 6a) were either absent or identified at low VAF after engraftment with a slight increase in VAF after VXL treatment (Fig. 6b, c; yellow, green and orange clusters). In contrast, SNVs identified at diagnosis showed only little change in VAF in the control PDXs but had low VAF upon VXL treatment (Fig. 6a–c; red cluster). To further examine these changes, clonal evolution was investigated in ALL-215 using PyClone, which identified five prominent sub-clones. These sub-clones were visualised using fishplots to compare the clonal structure observed from Dx to Rel in the patient, evolution from Dx to PDX-control and Dx to VXL-treated PDXs (Fig. 6d–f). The proposed evolutionary model highlighted sub-clonal changes occurring from Dx to Rel in patient ALL-215, which were more prevalent upon VXL treatment in the PDX, indicating that VXL treatment caused the expansion of a subclone representing ALL cells which drove relapse at a later timepoint in the patient’s disease course.

## Discussion

The results presented in this study describe the development of a PDX model for prediction of relapse in paediatric MR ALL patients. After initially establishing optimal engraftment conditions, 30 biopsies taken from MR ALL patients at diagnosis were each engrafted into six immune-compromised NSG mice to determine if such a PDX model was able to predict patient outcome prior to relapse. Although engraftment was usually consistent, variation within the group of six was seen for some sets, in accordance with previous reports in different cancers, including ALL.^21,33–35^ This variation has been discussed as a potential limitation of PDX models in the literature.^36^ While the heterogenous nature of ALL and presence of sub-clones can undoubtedly affect PDX engraftment,^36^ most reports highlight the strength of PDXs to retain original tumour characteristics and to represent the heterogeneity of the disease very well.^37^

The presented PDX model demonstrated the value of VXL chemotherapy in distinguishing between PDXs derived from MR ALL patients who either relapsed or not, suggesting that the intrinsic growth activity of leukaemia cells in PDXs under in vivo chemotherapy mimics the growth of ALL cells under such chemotherapeutic pressure in patients. This finding in MR ALL patients from the ANZCHOG ALL8 trial confirms a previous study showing that ALL PDXs treated with VXL chemotherapy reflected outcomes of a small and heterogeneous cohort of ALL patients treated under variable induction chemotherapy protocols.^26^

Statistical analysis of disease progression patterns in the VXL-treated mice revealed significant differences at TT25% between PDXs derived from CR1 compared to Rel patients. A similar trend was observed for TTL, which was not significant; in contrast to a study by Meyer et al.,^27^ where short TTL was indicative of early relapse. Key experimental differences, such as onset of ALL in PDXs, differences in the number of cells inoculated, differences in mouse strain (NOD/SCID versus NSG), shorter length of the monitoring period and number of PDXs excluded as non-engrafting may account for the different results of these two studies.

Statistical analysis of individual cytogenetic subgroups of patients (*ETV6-RUNX1*, Hyperdiploid and B-other) resulted in potential earlier predictive cut-off timepoints for patients harbouring the *ETV6-RUNX1* translocation. However, in our cohort, there were only six *ETV6-RUNX1* patients, and a larger study is required to make reliable conclusions.

Measuring MRD 15 days after the start of induction treatment is another feature that has been shown to improve prediction of patient relapse in MR-ALL.^12,14,38^ The value of day 15 MRD was demonstrated in a subset of 253 ANZCHOG ALL8 patients that include 22 of the 30 patients used for this PDX study.^14^ The cohort used in this study was too small and heavily pre-selected to make such a comparison possible, but an expanded cohort analysis may show that using a combination of the PDX predictive model and patient MRD at day 15 and day 33 could further improve relapse prediction in MR-ALL patients.

Interestingly, two CR1 patient biopsies (ALL-213 and ALL-214) showed strong disease progression despite VXL treatment, whereas one relapsed patient biopsy did not show progression upon VXL treatment (ALL-226; Fig. 5). The clinical data (Table 1) on these two patient samples showed in retrospect that they would have been classified as B-other ALL. Moreover, both presented with higher WCCs at diagnosis compared to most of the CR1 group (158.4 and 45.8, respectively). Patients with B-other ALL have poorer outcomes than ALL with either *ETV6-RUNX1* and Hyperdiploid > 50, which together constitute the majority of MR patients.^39,40^ High WCC (>50) is a well-established marker for poor patient outcome in the clinic,^41,42^ indicative of a more aggressive disease which is likely accountable for the observed strong engraftment of these samples. The rapid disease progression in these two CR1 patients may also be explained by the absence of doxorubicin in our VXL treatment regimen, which both patients would have received as part of ANZCHOG ALL8 induction therapy. No distinguishing features could be identified in the non-engrafting relapsed patient.

Extending our findings to a real-world setting, and assuming a true relapse rate of 20%,^5,9^ the 85.4% specificity and 66.7% sensitivity of the PDX model would translate to a positive predictive value of only 52% but a negative predictive value of 91%. The former might be considered insufficient evidence to convince patients, guardians and healthcare workers to intensify the treatment in an attempt to prevent relapse, while the latter might be used to reassure against the likelihood of relapse.

Mutational analysis of biopsies derived from four relapsed MR ALL patients and targeted sequencing of both original patient samples and PDXs demonstrated that the mutational landscape of the PDXs was largely consistent with the original diagnostic biopsy. This finding is in agreement with numerous studies, providing another validation that PDXs can reliably mimic both the molecular profile of the original tumours and the drug responses in various types of cancers, including leukaemia.^43–46^

The PDXs in this study were subjected to only one round of engraftment and treatment, resulting in very few molecular changes compared to diagnosis, in agreement with our previous work showing that multiple rounds of engraftment were necessary to observe clear molecular changes in PDXs.^23^ A more recent genomics study suggests that PDXs, upon serial transplantation, show greater deviations from their tumour of origin than initially believed.^47^ Such studies emphasise the importance of a model, such as ours, which can predict outcome quickly, avoiding serial transplantation and thus potential genomic changes induced by multiple engraftments.

Interestingly, one set of the molecularly characterised PDXs (ALL-215, Fig. 6 and Supplementary Fig. S7) exhibited the appearance of sub-clones that were identified in the patient’s relapse biopsy, highlighting the likely emergence of treatment-resistant clone(s). Further examination of the molecular profiles of the emergent PDXs, especially more transient processes such as gene expression and epigenetic changes,^48^ may provide even greater insight into the mechanisms of clonal selection and chemo-resistance in MR-ALL patients destined to relapse. Our PDX model might then serve as a platform to test potential treatment options to tailor the most effective therapies to the appropriate patients, ultimately leading to improved patient outcome. In summary, this study provides an important step towards the clinical application of ALL PDXs for MR ALL patient outcome prediction and, when combined with the assessment of MRD levels post treatment initiation, provides the basis for further work aiming to determine molecular signatures and potential biomarkers for refinement of the current prediction model.

## Supplementary information

Supplementary File Revision 1

Supplementary Table S1

Supplementary Table S2

Supplementary Table S3

Supplementary Table S4

Supplementary Table S5

Supplementary Table S6A

Supplementary Table S6B

## Data Availability

All data associated with this study are available from the authors upon request.
