# Supplementary material for: Examining treatment responses of diagnostic marrow in murine xenografts to predict relapse in children with acute lymphoblastic leukaemia
Source: Br J Cancer. 2020 Jun 15;123(5):742–51. doi: 10.1038/s41416-020-0933-4 (PMC7462974; doi:10.1038/s41416-020-0933-4)
Supplement: Supplementary file 1 — Supplementary File Revision 1 [file 41416_2020_933_MOESM1_ESM.docx]

**Examining treatment responses of diagnostic marrow in murine xenografts to predict relapse in children with acute lymphoblastic leukaemia**

**Running title:** Relapse prediction for medium risk ALL

Abdulmohsen M Alruwetei^1,2,*^, Katerina Bendak^1,*^, Babasaheb D Yadav^1^, Hernan Carol^1^, Kathryn Evans^1^, Chelsea Mayoh^1^, Rosemary Sutton^1^, Glenn M Marshall^1,3^and Richard B Lock^1^

^1^Children's Cancer Institute, School of Women’s and Children’s Health, UNSW Sydney, Sydney, Australia
^2^College of Applied Medical Science, Medical laboratory department, Qassim University, Qassim, Saudi Arabia
^3^Kids Cancer Centre, Sydney Children's Hospital, Randwick, Australia

*****These authors contributed equally to this work

**Corresponding author:**

Richard B Lock, PhD

Children’s Cancer Institute

PO BOX 81, Randwick, NSW 2031, Australia

[rlock@ccia.unsw.edu.au](mailto:rlock@ccia.unsw.edu.au)

Phone: +61 2 9385 2513 20 Fax: + 61 2 9662 6583

**Competing interests:** The authors declare that there are no competing interests.

**SUPPLEMENTARY MATERIAL**

**SUPPLEMENTARY METHODS**

**Whole genome sequencing:**

DNA was extracted from diagnostic, remission (day 147, MRD negative) and relapse bone marrow biopsies using TRIzol (Thermo Fisher Scientific) and subsequently sequenced on the Illumina HighSeq 2000/2500. Illumina paired-end whole genome sequencing data were aligned to the human genome assembly (build hg38) using BWA-mem (v0.7.11). Alignments were sorted and indexed with SAMtools (v. 1.3.1) and duplicates marked with Picard Tools (version 2.4.1, http://broadinstitute.github.io/picard). Indels were realigned and bases recalibrated using the Genome Analysis Toolkit (v3.6). Variants were called with Strelka (v1.0.15) and ANNOVAR was used to annotate the variants (refGene, dbnsfp30a, cosmic70, esp6500siv2_all, 1000g2015aug_all, 1000g2015aug_afr, 1000g2015aug_eas, 1000g2015aug_eur, 1000g2015aug_amr, 1000g2015aug_sas, avsnp147, clinvar_20160302). [^1-5^](#_ENREF_1). Genomic variants were filtered to ensure read quality (>10) and genome coverage (>30). Only SNVs previously described as detrimental/possibly detrimental in at least one database annotated through ANNOVAR were retained for further analysis (Supplementary Table S5) [^6^](#_ENREF_6).

**Targeted amplicon sequencing**

Targeted sequencing was conducted on DNA isolated using TRIzol from bone marrow biopsies collected at diagnosis, remission (day 147, MRD negative) and relapse from patients ALL-202, ALL-203, ALL-215 and ALL-217 and bone marrow or spleen samples from the corresponding PDXs. A total of 184 target sites were chosen *a priori* from mutations identified in the WGS data set (Supplementary Table S6), representing mutated sites found at diagnosis only, relapse only or present in both biopsies for all four patients. Primers targeting genes of interest were prepared using the AmpliSeq targeted enrichment system (Supplementary Table S6B; Illumina, Australia) and paired-end sequencing was conducted using MiSeq v2 (Ramaciotti Centre for Genomics, Australia) to an average depth of >1,000 reads per site. Data analysis utilized the same pipeline mentioned above with an additional in-house python script to calculate variant allele frequencies (VAFs; Supplementary Table S6A).

Sub-clonal distribution was assessed using PyClone (v0.13.1) [^7^](#_ENREF_7), followed by validation and inference of likely evolutionary models using the R (v3.5.1) [^8^](#_ENREF_8) package clonevol (v0.99.11) [^9^](#_ENREF_9). The proposed evolutionary model based on the five most prominent sub-clones was visualized using the R package fishplot (v0.4) [^10^](#_ENREF_10).

**SUPPLEMENTARY FIGURES**

**
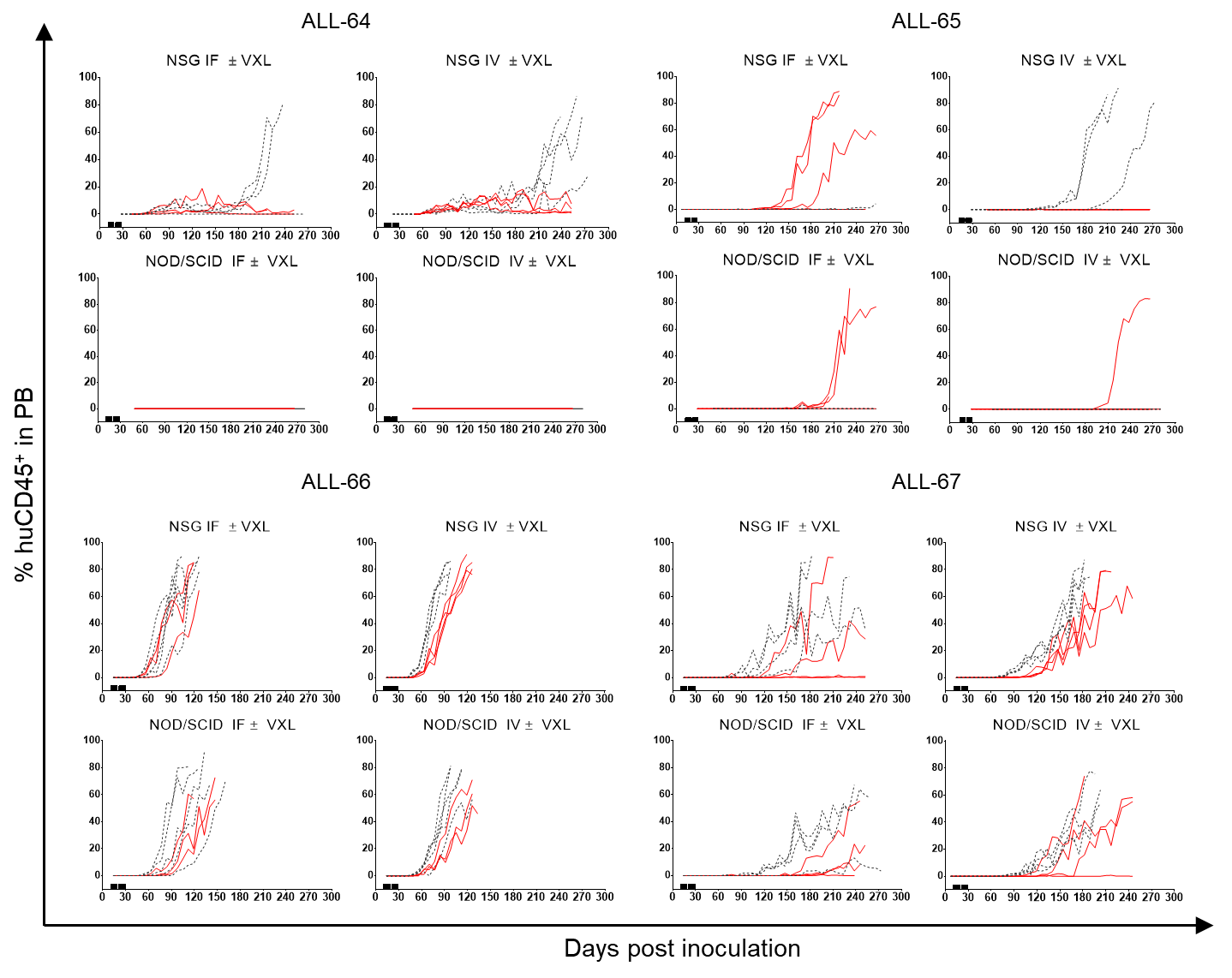
**

**Supplementary Figure S1. Engraftment of MR ALL patient samples subjected to divergent engraftment strategies.** Graphs show the progression of patient cells over the monitoring period (up to 280 days) in the PB of NSG and NOD/SCID mice used to establish ALL-64, ALL-65, ALL-66 and ALL-67. Each line indicates the percentage of human CD45^+^ cells in the PB of a single mouse of VXL-treated (red line) and control (black, dashed line) mice over time. The solid black squares indicate the VXL treatment period.

**
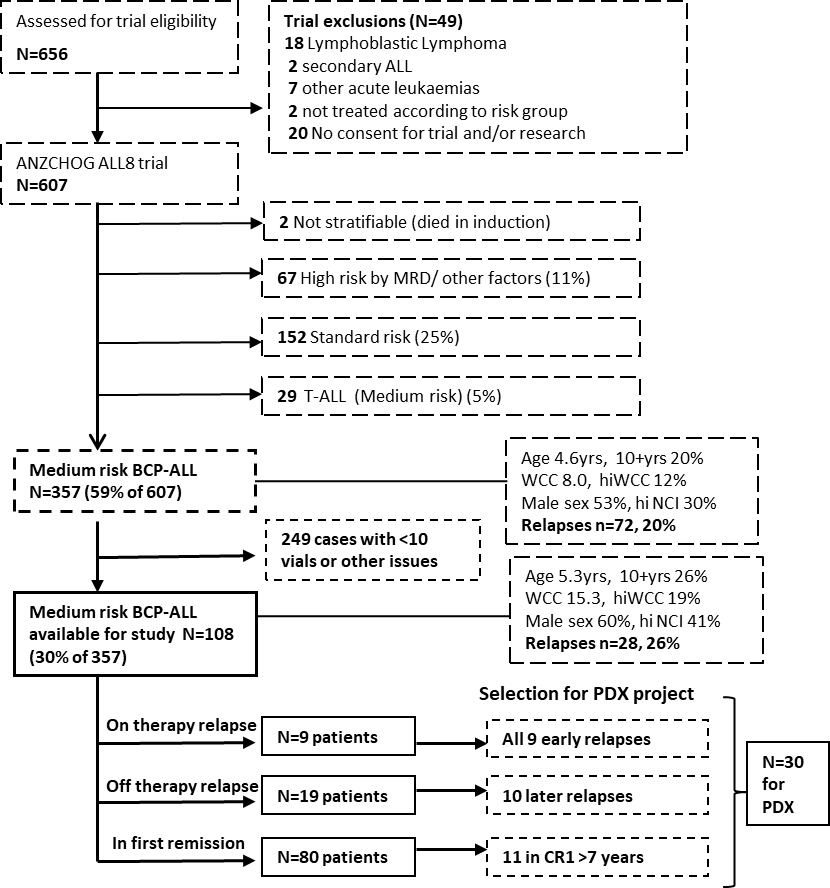
**

**Supplementary Figure S2. Selection of MR ALL patient samples for this study.** The diagram shows the sequential selection of 30 patients for this xenografting study from an initial cohort of 656 patients assessed for eligibility in the ANZCHOG ALL8 trial. Patients were assessed according to standard, medium and high-risk factors, with 30 uniformly treated medium-risk BCP-ALL patients selected for engraftment. Key characteristics are shown according to trial-defined standard-, medium- and high-risk groups. Age is mean age at diagnosis.

**Supplementary Figure S3. Assessing the reproducibility of the MR ALL PDX model.** Graphs show the engraftment patterns of three patient biopsies used in both, the initial study and combined with all other samples, to establish ALL-64/202 (A and B), ALL-65/220 (C and D) and ALL-66/215 (E and F).


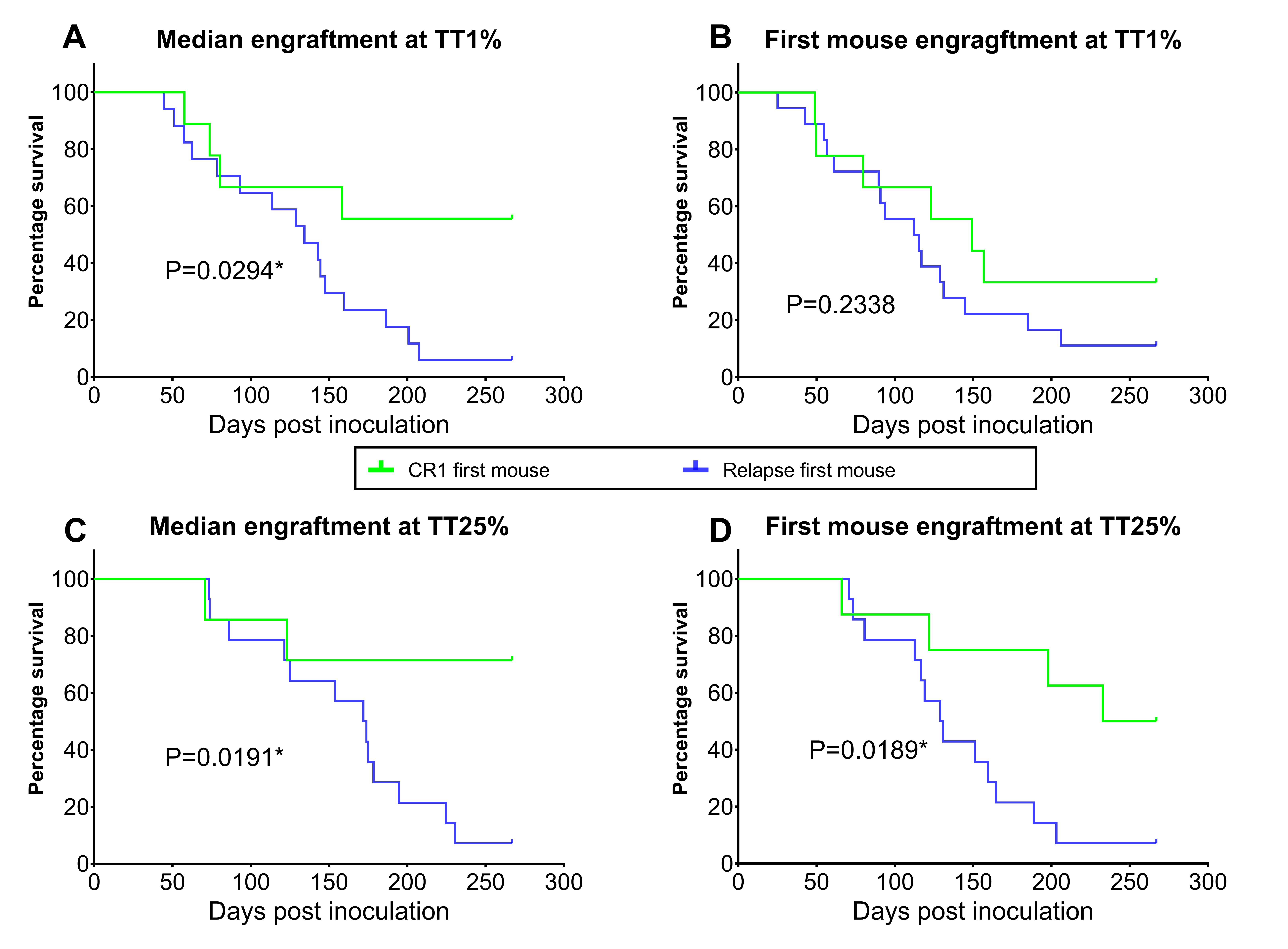


Supplementary Figure S4. Comparison of the EFS of MR-ALL patient samples based on the median engraftment or the engraftment of the first mouse

**.** The EFS of VXL treated mice inoculated with samples from each patient outcome group (CR1 or Rel) was compared either as the median of each group (A, C) or the first mouse to engraft (B, D). Events were defined as TT1% (A, B) or TT25% (C, D). Mouse EFS was plotted by Kaplan-Meier curves and compared for statistical significance using the log-rank test.

**Supplementary Figure S5. ROC analysis of all engraftment events to determine outcome predictive cut-off.** ROC analysis was conducted to determine the optimal criteria predicting patient outcome for the first mouse reaching TT1% (A), the median mouse reaching TT1% (B) or TT25% (C), for the first mouse reaching TTL (D) or the median mouse reaching TTL (E). Kaplan-Meier graphs show the mouse EFS in the MR-ALL cohort based on classifiers determined by ROC analysis of the first mouse reaching TT1% prior to 144.7 days (F), the median mouse reaching TT1% prior to 207.6 days (G) or TT25% prior to 230.6 days (H), the first mouse reaching TTL prior to 201 days (I) or the median mouse reaching TTL prior to 239 days (J). The log-rank test was used to analyse statistical differences in length of engraftment between the two subgroups of patients.

**A**

**B**

**C**

**D**

**F**

**E**

**Supplementary Figure S6 (A-F). Comparison of the relapse predictive power of the PDX model with standard methods of risk prediction.** The relapse-free survival (RFS) was plotted as % probability from time of diagnosis. Patients were stratified either using the PDX model or more standard methods of risk prediction. (A) patients from Figure 5 were stratified according to the median TT25% of < or > 248 days (ALL-207 was excluded from analysis since only one VXL treated mouse reached 128 days post inoculation). (B-I) All 30 patients were stratified according to NCI risk criteria (B), MRD status (C), *ETV6-RUNX1* (D), ALL favourable versus intermediate risk genetics (E), IKZF1plus status (F), Ph-like ALL status (G), IKZF1 deletion alone (H), and IKZF1 or CRLF2 deletions (I). Data were analysed by log-rank (Mantel Cox) test and Kaplan-Meier plots. NS, not significant.

**G**

**I**

**H**

**Supplementary Figure S6 (G-I). Comparison of the relapse predictive power of the PDX model with standard methods of risk prediction.** The relapse-free survival (RFS) was plotted as % probability from time of diagnosis. Patients were stratified either using the PDX model or more standard methods of risk prediction. (A) patients from Figure 5 were stratified according to the median TT25% of < or > 248 days (ALL-207 was excluded from analysis since only one VXL treated mouse reached 128 days post inoculation). (B-I) All 30 patients were stratified according to NCI risk criteria (B), MRD status (C), *ETV6-RUNX1* (D), ALL favourable versus intermediate risk genetics (E), IKZF1plus status (F), Ph-like ALL status (G), IKZF1 deletion alone (H), and IKZF1 or CRLF2 deletions (I). Data were analysed by log-rank (Mantel Cox) test and Kaplan-Meier plots. NS, not significant.

**
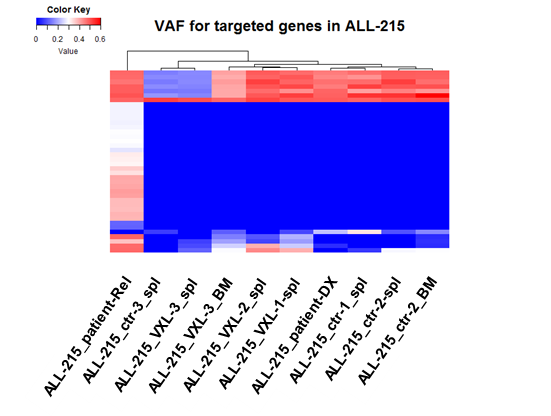
**

**Supplementary Figure S7. Mutational analysis of patient BM aspirates and corresponding vehicle and VXL treated PDXs.** AmpliSeq was conducted on patient samples and corresponding PDXs and resulting VAFs are shown as a heatmap. VAFs of SNPs analysed using targeted sequencing were compared between Dx, Rem and Rel patient biopsies and control and VXL treated PDX samples.

**REFERENCES**

1. Li H, Durbin R. Fast and accurate short read alignment with Burrows-Wheeler transform. *Bioinformatics* 25, 1754-1760 (2009)

2. McKenna A, Hanna M, Banks E, Sivachenko A, Cibulskis K, Kernytsky A*, et al.* The Genome Analysis Toolkit: a MapReduce framework for analyzing next-generation DNA sequencing data. *Genome Res* 20, 1297-1303 (2010)

3. Li H, Handsaker B, Wysoker A, Fennell T, Ruan J, Homer N*, et al.* The Sequence Alignment/Map format and SAMtools. *Bioinformatics* 25, 2078-2079 (2009)

4. DePristo MA, Banks E, Poplin R, Garimella KV, Maguire JR, Hartl C*, et al.* A framework for variation discovery and genotyping using next-generation DNA sequencing data. *Nat Genet* 43, 491-498 (2011)

5. Saunders CT, Wong WS, Swamy S, Becq J, Murray LJ, Cheetham RK. Strelka: accurate somatic small-variant calling from sequenced tumor-normal sample pairs. *Bioinformatics* 28, 1811-1817 (2012)

6. Wang K, Li M, Hakonarson H. ANNOVAR: functional annotation of genetic variants from high-throughput sequencing data. *Nucleic Acids Res* 38, e164 (2010)

7. Roth A, Khattra J, Yap D, Wan A, Laks E, Biele J*, et al.* PyClone: statistical inference of clonal population structure in cancer. *Nat Methods* 11, 396-398 (2014)

8. Dessau RB, Pipper CB. [''R"--project for statistical computing]. *Ugeskr Laeger* 170, 328-330 (2008)

9. Dang HX, White BS, Foltz SM, Miller CA, Luo J, Fields RC*, et al.* ClonEvol: clonal ordering and visualization in cancer sequencing. *Ann Oncol* 28, 3076-3082 (2017)

10. Miller CA, McMichael J, Dang HX, Maher CA, Ding L, Ley TJ*, et al.* Visualizing tumor evolution with the fishplot package for R. *BMC Genomics* 17, 880 (2016)
